# Application effect of an evidence-based early functional exercise program in kidney transplant recipients

**DOI:** 10.1097/MD.0000000000051161

**Published:** 2026-10-30

**Authors:** Miao Zhang, Peili Shi, Xiaowei Su, Ying Wang, Shuwen Li

**Affiliations:** a Department of Nursing, The Second Affiliated Hospital of Anhui Medical University, Hefei, Anhui, China; b Taikang Health Industry Research Institute, Anhui Medical University, Hefei, Anhui, China; c Department of Urology, The Second Affiliated Hospital of Anhui Medical University, Hefei, Anhui, China; d School of Nursing, Anhui Medical University, Hefei, Anhui, China.

**Keywords:** early functional exercise, evidence-based nursing, kidney transplantation, postoperative rehabilitation, retrospective study

## Abstract

This study evaluates the clinical application effect of an evidence-based early functional exercise program in kidney transplant recipients. A retrospective study design was adopted. A total of 84 patients who underwent kidney transplantation in a tertiary hospital from January 2025 to March 2026 were selected as the study subjects. According to whether they received the evidence-based early functional exercise program, they were divided into a control group (conventional care) and an evidence-based practice (EBP) group (evidence-based program care), with 42 cases in each group. Propensity score matching was used to ensure baseline comparability between the 2 groups. Early functional recovery indicators, activities of daily living, pain scores, self-efficacy, and postoperative complication rates were compared between the 2 groups. The time to 1st postoperative ambulation in the EBP group was significantly earlier than that in the control group (median 23.0 vs 98.0 hours, *P* < .001), and the rate of ambulation within 24 hours was significantly higher (66.7% vs 7.1%, *P* < .001). The time to 1st flatus, time to 1st defecation, and postoperative hospital stay in this group were significantly better than those in the control group (*P* < .001). At discharge, the Barthel index score (88.2 ± 5.9 vs 75.8 ± 7.6, *P* < .001) and the proportion of patients with complete independence in daily living (47.6% vs 14.3%, *P* = .005) in the EBP group were significantly higher than those in the control group. At 3 days postoperatively, the numeric rating scale score in the EBP group was lower than that in the control group (median 3.5 vs 5.0, *P* < .001); the improvement in self-efficacy score was also significantly better than that in the control group (*P* < .001). In this retrospective propensity score-matched cohort, receipt of the evidence-based early functional exercise program was associated with earlier postoperative ambulation and gastrointestinal recovery, better activities of daily living and self-efficacy, lower pain scores, and a lower observed complication rate, without an observed increase in falls. These findings suggest that the program may have potential clinical value, although prospective multicenter studies are needed to confirm causality and generalizability.

## 1. Introduction

Kidney transplantation (KT) is widely recognized as the optimal treatment for patients with end-stage renal disease, offering significantly better long-term survival and quality of life compared to long-term maintenance hemodialysis or peritoneal dialysis.^[1]^ With continuous advancements in surgical techniques, perioperative management, and immunosuppressive regimens, short-term graft survival after KT has greatly improved.^[2]^ However, the early postoperative period remains a critical phase during which patients face risks of various complications, including delayed graft function, infection, gastrointestinal dysfunction, and various adverse events associated with prolonged bed rest.^[3,4]^

Prolonged bed rest is closely associated with numerous adverse outcomes, such as muscle atrophy, decreased cardiopulmonary function, venous thromboembolism, and delayed physical function recovery.^[5,6]^ For KT recipients, these issues are of particular concern because they may prolong hospital stay, increase medical costs, and negatively affect long-term prognosis.^[7]^ Early functional exercise, as a core component of the enhanced recovery after surgery concept, aims to counteract the adverse effects of immobilization and accelerate the postoperative rehabilitation process.^[8,9]^

Previous studies have confirmed that early activity after KT can improve muscle strength, reduce the incidence of deep vein thrombosis and pulmonary infection, and shorten hospital stay. Randomized controlled trials have reported that early activity interventions can significantly improve physical function and reduce postoperative complications in KT recipients.^[10]^ Systematic reviews have also concluded that exercise training initiated early after surgery helps improve patients’ cardiorespiratory fitness and muscle strength. Despite these encouraging findings, the implementation of early functional exercise in clinical practice remains highly variable. Many transplant centers still rely on traditional, non-standardized approaches, where exercise is often delayed or guided solely by clinical experience, lacking evidence-based standardized protocols.

Currently, there is no consensus on the optimal timing, intensity, frequency, or progression criteria for early functional exercise after KT. Studies on systematic, phased exercise programs based on high-level evidence and evaluations of their clinical effects are relatively limited.^[10]^ Moreover, research comparing multidimensional outcomes between evidence-based structured exercise programs and conventional care is also uncommon. Furthermore, concerns among some healthcare professionals that early activity may increase risks such as graft injury, incision bleeding, or hemodynamic instability have, to some extent, hindered the dissemination of such programs.

Therefore, this study aimed to evaluate an evidence-based early functional exercise program after KT and to compare early functional recovery indicators, activities of daily living (ADL), pain levels, self-efficacy, and complication rates between patients receiving the program and those receiving conventional care. Given the retrospective design, the study focused on associations between program receipt and clinical outcomes. We hypothesized that receipt of the evidence-based early functional exercise program would be associated with earlier postoperative recovery, better physical and psychological outcomes, and lower complication rates without an observed increase in safety events.

## 2. Methods

### 2.1. Study design

This study was approved by the Ethics Committee of Anhui Medical University (approval 2026-AHMU-0187). This retrospective cohort study included patients who met the inclusion and exclusion criteria and underwent allogeneic KT in the Department of Urology of our hospital from January 2025 to March 2026. Patients treated before the implementation of the evidence-based program comprised the conventional-care cohort, whereas patients treated after its implementation and who received the program comprised the evidence-based practice (EBP) cohort. Because the 2 groups were drawn from different chronological periods, temporal confounding arising from potential changes in surgical care, staffing, perioperative management, or other unmeasured clinical practices could not be completely excluded and was therefore considered when interpreting the findings. To reduce measured baseline imbalance, propensity score matching (PSM) was used to perform 1:1 matching between the 2 groups. Finally, 84 patients were included in the matched analysis, with 42 patients in each group.

### 2.2. Inclusion and exclusion criteria

#### 2.2.1. Inclusion criteria

First-time allogeneic KT, with the surgical procedure being either open or laparoscopic-assisted KT; age ≥18 years, male or female; postoperative conscious state, stable vital signs, and basic language comprehension and communication abilities; postoperative hospital stay ≥7 days to ensure a sufficient observation period for evaluating the effects of functional exercise; and complete clinical data, including postoperative nursing records, rehabilitation exercise records, discharge summaries, and other key information that could be traced.

#### 2.2.2. Exclusion criteria

Multiorgan transplantation (e.g., combined kidney-pancreas transplantation and combined kidney-liver transplantation); presence of limb movement disorders, severe musculoskeletal diseases, neurological diseases, or other comorbidities affecting normal activity before surgery; presence of severe cardiac, pulmonary, or hepatic insufficiency or hemodynamic instability precluding tolerance of early functional exercise; occurrence of severe postoperative complications (e.g., nonfunctioning renal graft, renal graft rupture, severe active bleeding, septic shock, and reoperation) leading to interruption or inability to implement the functional exercise plan; postoperative inability to comply with functional exercise instructions due to mental disorders, cognitive dysfunction, or other reasons; missing key variables in clinical data (e.g., time to 1st ambulation, exercise records, and outcome indicators) affecting data integrity.

### 2.3. Sample size calculation and propensity score matching

#### 2.3.1. Propensity score matching

To maximize control over confounding bias and ensure baseline comparability between the 2 groups, this study used PSM to perform 1:1 matching between the control group and the EBP group. Matching variables included age, sex, body mass index (BMI), primary disease type, cold ischemia time, donor kidney source, and preoperative comorbidities. The specific steps were as follows: First, a logistic regression model was constructed with the group variable as the dependent variable and the above variables as covariates to calculate the propensity score. Second, the nearest neighbor matching method (caliper value set at 0.02) was used to match each patient in the EBP group with a control group patient with the closest propensity score. Finally, balance tests were performed on the baseline data of the 2 groups after matching. After matching, a total of 84 patients were successfully included, with 42 patients in each of the control group and the EBP group. There were no statistically significant differences in baseline data between the 2 groups (*P* > .05), indicating good comparability.

#### 2.3.2. Sample size determination

Because this was a retrospective cohort study, the sample size was determined by the number of eligible patients available during the predefined study period and the number retained after 1:1 PSM, rather than by an a priori power calculation. After application of the eligibility criteria and PSM, 84 patients (42/group) were included in the final analysis.

### 2.4. Evaluation of nursing methods in the 2 groups

#### 2.4.1. Nursing method in the control group

Patients in the control group received routine postoperative nursing care and functional exercise guidance from the department. After awakening from anesthesia, the responsible nurse instructed patients to perform bedside ankle dorsiflexion-plantarflexion exercises and ankle pump exercises, with each session lasting 5 to 10 minutes, once every 2 hours. Simultaneously, patients were instructed to perform deep breathing exercises 3 to 4 times/d, with 5 to 8 deep breaths each time. On postoperative day 2, the head of the bed was raised to 30° to 45° to assist patients into a semi-recumbent position, maintained for 15 to 20 minutes each time, 3 times/d; the duration could be gradually extended as tolerated. On postoperative day 3, patients were guided to perform sitting-up exercises in bed with assistance, gradually transitioning from passive to active sitting-up. On postoperative day 7, under the premise of confirmed stable vital signs, no active bleeding, and good wound healing, the patient was assisted by a nurse or family member to ambulate for the 1st time. The patient 1st sat at the bedside for 3 to 5 minutes, then, if no dizziness or other discomfort was present, was assisted to stand at the bedside for 2 to 3 minutes, and subsequently attempted slow walking. The initial walking distance generally did not exceed 10 m, and the activity time was controlled within 10 to 15 minutes, 1 to 2 times/d. Subsequent activity levels were increased as appropriate based on the patient’s subjective tolerance, with no clear daily increment standards. Guidance on functional exercise was primarily delivered through verbal education by the responsible nurse, supplemented by printed health education sheets. The nurse decided whether to increase activity based on the patient’s subjective reports of fatigue level, pain score, and other subjective feelings. No standardized assessment tools, activity intensity scales, or progression criteria were used. Monitoring of vital signs during exercise was nonmandatory and was primarily self-observed by the patient or family members.

#### 2.4.2. Nursing method in the EBP group

Patients in the EBP group received, in addition to routine nursing care, the strictly implemented Early Functional Exercise Program for patients after KT, which was previously developed using evidence-based methods. This program, developed by an evidence-based nursing practice team based on the best available evidence, was initiated 24 hours postoperatively and continued until discharge. It consisted of 3 phases, each specifying activity content, frequency/intensity, progression criteria, and safety measures, as detailed below.

##### 2.4.2.1. Initiation timing assessment

Postoperatively, the responsible nurse conducted a systematic assessment using the “Early Activity Initiation Assessment Form.” The in-bed functional exercise was initiated within 24 hours postoperatively when the patient met all of the following criteria: awake and alert, with a Richmond agitation-sedation scale score ≥−2 (level 5, grade A); hemodynamically stable, requiring no vasoactive medications to maintain blood pressure and with a mean arterial pressure ≥65 mm Hg (level 5, grade A); no signs of active bleeding, with drainage volume <50 mL/h and stable hemoglobin (level 5, grade A); numeric rating scale (NRS) pain score ≤4 or pain tolerable after analgesic treatment (level 1c, grade A); no severe gastrointestinal reactions such as nausea or vomiting (level 5, grade A). If any criterion was not met, initiation was postponed, and reassessment was performed every 4 hours until the criteria were met.

##### 2.4.2.2. Phased progressive exercise protocol

###### Phase 1: In-bed activity period (postoperative 24–72 hours)

Ankle pump exercises: 10 to 15 repetitions per set, 6 to 8 sets/d, holding each dorsiflexion/plantarflexion position for 3 to 5 seconds (level 1a, grade A); quadriceps isometric contractions: 10 to 15 repetitions per set, 6 to 8 sets/d, holding each contraction for 5 to 10 seconds (level 1c, grade A); straight leg raises: 10 to 15 repetitions per set, 6 to 8 sets/d, lifting the leg 15 to 20 cm off the bed and holding for 5 seconds (level 1c, grade A); and deep breathing exercises: 10 to 20 abdominal breaths with pursed-lip exhalation per hour (level 1a, grade A). Exercise intensity was guided by a heart rate increase not exceeding 20 beats/min above resting heart rate or a rating of perceived exertion score ≤11 (level 1c, grade A). Progression to phase 2 occurred after 24 consecutive hours without reports of discomfort and the ability to independently complete the full set of in-bed exercises.

###### Phase 2: Bedside adaptation period (postoperative 48 hours—7 days)

Bedside sitting training: initial sitting duration ≥5 minutes, increasing by 5 to 10 minutes daily until able to sit independently for 30 minutes, 2 to 3 times/d (level 1a, grade A); unsupported seated balance training at the bedside: weight-shift training performed in the sitting position, 5 to 10 minutes/session, 2 to 3 times/d (level 1c, grade A); and bedside standing training: starting at 30 seconds, increasing by 15 to 30 seconds each time, 3 to 5 times/d (level 1c, grade A). Monitoring for dizziness, pain in the graft kidney area, and blood pressure fluctuations was performed throughout (level 5, grade A). Progression to phase 3 occurred when the patient could sit independently at the bedside for 30 minutes without orthostatic hypotension and could stand with assistance for ≥3 minutes.

###### Phase 3: Progressive ambulation period (postoperative day 5 to discharge)

Bedside walking training: daily step goal starting at 100 steps, increasing by 100 to 200 steps/d, with a target of 1000 to 1500 steps/d before discharge (level 1a, grade A); in-ward walking training: walking duration gradually extended from 5 to 20 to 30 minutes/session, 2 to 3 times/d (level 1a, grade A); low-resistance elastic band resistance training: 2 times/d, 10 to 15 minutes/session (level 1c, grade A); and exercise intensity controlled within a rating of perceived exertion score of 11 to 14 (level 1c, grade A). Before discharge, patients and their families were provided with the “Home Functional Exercise Guidance Manual,” which clearly outlined the post-discharge exercise plan (level 3, grade A).

##### 2.4.2.3. Safety assurance and monitoring measures

Within 5 minutes before and after each exercise session, the responsible nurse monitored and recorded the patient’s blood pressure, heart rate, respiratory rate, and oxygen saturation (level 1c, grade A). During exercise, a nurse or trained family member provided bedside supervision throughout. The Morse fall scale was used for assessment once daily; patients with a score ≥45 were considered at high risk of falling and required enhanced protective measures (level 2, grade A). Clear termination criteria were established. Exercise was stopped immediately and the physician was notified if any of the following occurred: systolic blood pressure <90 or >180 mm Hg, heart rate <50 or >120 bpm, oxygen saturation <92%, patient complaints of chest pain, dyspnea, dizziness, or severe incisional pain, fresh exudate on the incisional dressing, or sudden increase in drainage volume (level 5, grade A).

##### 2.4.2.4. Multidisciplinary collaboration and documentation management

A multidisciplinary team was established, consisting of the responsible nurse, attending physician, and rehabilitation nurse who had received unified training. After the morning meeting each day, the team jointly reviewed the patient’s exercise performance from the previous day and decided on the activity goals and protocol adjustments for the current day (level 3, grade A). All exercise content, duration, intensity, vital signs, and patient responses were systematically documented in the “Early Functional Exercise Implementation Record Form,” which served as the basis for daily assessment and progression (level 5, grade A). During each shift handoff, the responsible nurse highlighted the patient’s current exercise phase, completion status, and existing problems to ensure continuity of the intervention (level 5, grade A).

### 2.5. Data collection

#### 2.5.1. Collection of baseline data

A self-designed patient baseline information form was used to collect demographic data (age, sex, BMI, marital status, occupation, education level, and type of medical insurance) and disease-related data (primary disease type, preoperative dialysis modality and duration, donor kidney source, cold ischemia time, and preoperative comorbidities) from the 2 groups via the hospital’s electronic medical record system. Data were independently extracted and verified by 2 researchers. Collection was performed after patient admission and before surgery.

#### 2.5.2. Collection of early functional recovery indicators

By reviewing postoperative nursing records and rehabilitation exercise records, the time to 1st postoperative ambulation, the rate of ambulation within 24 hours, the time to 1st flatus, the time to 1st defecation, and the postoperative hospital stay were collected. These indicators were recorded in real time by the responsible nurse, and the researchers compiled them uniformly after patient discharge.

#### 2.5.3. Collection of ADL data

The Barthel index (BI) was used to assess the ADL of patients in the 2 groups, with assessments performed preoperatively, on postoperative day 3, and on the day of discharge. This scale contains 10 items, with a total score ranging from 0 to 100; a higher score indicates better independence. Based on the score, independence was classified into 4 levels: 0 to 40 as severe dependence, 41 to 60 as moderate dependence, 61 to 99 as mild dependence, and 100 as complete independence. Assessments were completed at the bedside by uniformly trained researchers.

#### 2.5.4. Collection of pain scores

The NRS combined with the revised faces pain scale was used to assess pain intensity. Assessment time points were preoperatively, on postoperative day 3, and on the day of discharge. The NRS used a 0 to 10 point scale, with patients providing self-reported scores; the faces pain scale assisted in expressing pain perception, and the 2 were used together to improve assessment accuracy.

#### 2.5.5. Collection of self-efficacy data

The Chinese version of the general self-efficacy scale was used to assess self-efficacy levels. Assessments were performed preoperatively, on postoperative day 3, and on the day of discharge. This scale contains 10 items, using a 4-point scoring method, with a total score ranging from 10 to 40; a higher score indicates stronger self-efficacy. Assessments were conducted by the researcher while the patient was in a quiet state.

#### 2.5.6. Collection of complication data

By reviewing nursing records, progress notes, and medical order sheets during the postoperative hospital stay, the occurrence of complications including nausea and vomiting, abdominal distension, urinary tract infection, urinary retention, pulmonary infection, falls, and unplanned extubation was collected. Each complication was independently confirmed by 2 attending physicians. The overall complication rate was defined as the proportion of patients who experienced at least one of the listed postoperative complications; a patient with >1 complication was counted once in the overall rate.

### 2.6. Statistical analysis

Statistical Product and Service Solutions 26.0 statistical software was used for data analysis. First, normality tests (Shapiro–Wilk test) were performed for all continuous variables. Measurement data conforming to a normal distribution were expressed as mean ± standard deviation (*x̄* ± *s*), and an independent samples *t* test was used for comparison between groups. Measurement data not conforming to a normal distribution were expressed as median (interquartile range) [M (P25, P75)], and the Mann–Whitney *U* test was used for comparison between groups. Count data were expressed as number of cases (percentage), and comparison between groups was performed using the χ^2^ test; Fisher exact test was used when the theoretical frequency was <5. The comparison of ordinal data (e.g., independence level classification) between groups was also performed using the χ^2^ test. A *P* value <.05 was considered statistically significant. All statistical analyses were performed on the propensity score-matched sample to ensure baseline comparability between the 2 groups.

## 3. Results

### 3.1. Balance test of study subjects after matching

Propensity score matching was performed to reduce measured baseline imbalance between the 2 groups. As shown in Table 1, after matching, the 42 patients in the control group and the 42 patients in the EBP group were balanced in terms of demographic characteristics including age, sex, and BMI (*P* > .05). There were also no statistically significant differences in primary disease type, preoperative dialysis modality and duration, donor kidney source, cold ischemia time, or preoperative comorbidities including hypertension and diabetes (*P* > .05). These findings indicate improved baseline comparability after matching; however, residual confounding from unmeasured factors cannot be excluded.

**Table 1:** Comparison of baseline data between the 2 groups.

Item	Control group (n = 42)	Evidence-based practice group (n = 42)	Statistical value	*P* value
Demographic data				
Age (yr, *x̄* ± *s*)	46.5 ± 10.8	44.2 ± 11.5	*t* = 0.958	.341
Male, n (%)	26 (61.9)	30 (71.4)	χ^2^ = 0.624	.430
Body mass index, *M* (P25, P75)	24.0 (22.0, 25.9)	22.8 (20.6, 24.5)	*Z* = −1.745	.081
Married, n (%)	40 (95.2)	38 (90.5)	χ^2^ = 0.455	.500
Employed, n (%)	20 (47.6)	23 (54.8)	χ^2^ = 0.193	.660
Junior high school/high school or above, n (%)	33 (78.6)	36 (85.7)	χ^2^ = 0.936	.626
Urban employee medical insurance, n (%)	24 (57.1)	28 (66.7)	χ^2^ = 0.840	.359
Disease-related data				
Chronic glomerulonephritis, n (%)	25 (59.5)	22 (52.4)	χ^2^ = 0.432	.511
Preoperative hemodialysis, n (%)	36 (85.7)	32 (76.2)	χ^2^ = 1.246	.264
Preoperative dialysis duration (yr), *M* (P25, P75)	3.0 (1.0, 5.0)	2.0 (1.0, 3.3)	*Z* = −1.342	.180
Deceased donor kidney, n (%)	24 (57.1)	29 (69.0)	χ^2^ = 1.200	.273
Cold ischemia time (h, *x̄* ± *s*)	7.9 ± 2.2	8.3 ± 2.4	*t* = −0.801	.425
Preoperative comorbidities, n (%)				
Hypertension	25 (59.5)	28 (66.7)	χ^2^ = 0.459	.498
Diabetes mellitus	13 (31.0)	9 (21.4)	χ^2^ = 0.976	.323
Coronary heart disease	5 (11.9)	2 (4.8)	χ^2^ = 1.424	.233

### 3.2. Overall improvement in postoperative rehabilitation process

Table 2 shows that the median time to 1st postoperative ambulation was approximately 75 hours earlier in the EBP group than in the control group (23.0 vs 98.0 hours), and the proportion ambulating within 24 hours was higher (66.7% vs 7.1%, *P* < .001). The times to 1st flatus and 1st defecation were also earlier in the EBP group (*P* < .001). Postoperative hospital stay was shorter in the EBP group (12.0 ± 2.5 vs 16.5 ± 3.2 days, *P* < .001). These findings represent between-group associations in the matched retrospective cohort.

**Table 2:** Comparison of early functional recovery indicators between the 2 groups.

Indicator	Control group (n = 42)	Evidence-based practice group (n = 42)	Statistical value	*P* value
Time to 1st postoperative ambulation (h, *M* (P25, P75))	98.0 (84.5, 112.0)	23.0 (20.5, 30.5)	*Z* = −6.845	<.001
Rate of ambulation within 24 h, n (%)	3 (7.1)	28 (66.7)	χ^2^ = 31.304	<.001
Time to 1st flatus (h, *M* (P25, P75))	68.0 (56.0, 80.0)	42.0 (36.0, 52.0)	*Z* = −6.321	<.001
Time to 1st defecation (h, *M* (P25, P75))	92.0 (78.0, 108.0)	65.0 (56.0, 78.0)	*Z* = −5.847	<.001
Postoperative hospital stay (d, *x̄* ± *s*)	16.5 ± 3.2	12.0 ± 2.5	*t* = 7.117	<.001

### 3.3. Activities of daily living outcomes

The recovery of postoperative ADL is an important indicator for measuring rehabilitation quality. As shown in Table 3, the preoperative BI scores of the 2 groups were similar and at the same level. However, at 3 days postoperatively, the independence score of the control group decreased significantly, whereas the decrease in the EBP group was smaller, with a difference of 16.2 points between the 2 groups (*P* < .001). This gap widened further at discharge, with the EBP group achieving a score of 88.2, approaching the normal level. Notably, at discharge, nearly half (47.6%) of the patients in the EBP group achieved complete independence in daily living, whereas only 14.3% of the control group reached this level (χ^2^ = 12.905, *P* = .005).

**Table 3:** Comparison of activities of daily living between the 2 groups.

Indicator	Control group (n = 42)	Evidence-based practice group (n = 42)	Statistical value	*P* value
Barthel index score (points, *x̄* ± *s*)				
Pre-intervention (preoperative)	85.6 ± 8.2	84.2 ± 9.1	*t* = 0.744	.459
Postoperative day 3	52.3 ± 10.5	68.5 ± 9.8	*t* = −7.341	<.001
Day of discharge	75.8 ± 7.6	88.2 ± 5.9	*t* = −8.285	<.001
Independence level on day of discharge, n (%)			χ^2^ = 12.905	.005
Severe dependence (0–40)	2 (4.8)	0 (0)		
Moderate dependence (41–60)	8 (19.0)	2 (4.8)		
Mild dependence (61–99)	26 (61.9)	20 (47.6)		
Complete independence (100)	6 (14.3)	20 (47.6)		

### 3.4. Postoperative pain outcomes

In clinical practice, some healthcare professionals are concerned that early activity may exacerbate postoperative pain. In this study, the observed pain scores did not support a higher pain burden in the EBP group. There was no difference in preoperative NRS scores between the 2 groups. Although scores increased in both groups at 3 days postoperatively, pain scores were lower in the EBP group than in the control group (median 3.5 vs 5.0, *P* < .001). By discharge, the median pain score was 1.0 in the EBP group and 2.0 in the control group (Table 4).

**Table 4:** Comparison of pain scores between the 2 groups.

Indicator	Control group (n = 42)	Evidence-based practice group (n = 42)	Statistical value	*P* value
NRS score (points, *M* (P25, P75))				
Pre-intervention (preoperative)	1.0 (0, 2.0)	1.0 (0, 2.0)	*Z* = −0.218	.827
Postoperative day 3	5.0 (4.0, 6.0)	3.5 (3.0, 5.0)	*Z* = −4.652	<.001
Day of discharge	2.0 (1.0, 3.0)	1.0 (0, 2.0)	*Z* = −3.842	<.001

### 3.5. Self-efficacy outcomes

The patient’s belief in their own ability to recover, i.e., self-efficacy, directly affects their initiative and adherence to functional exercise. Table 5 presents the dynamic changes in general self-efficacy scale scores between the 2 groups. Preoperative scores were comparable between the 2 groups. At 3 days postoperatively, the self-efficacy score in the control group showed a significant decline (from 24.5 to 20.6), which may be related to postoperative experiences such as pain and fatigue. In contrast, the score in the EBP group increased slightly from the preoperative level at 3 days postoperatively (26.2). At discharge, the gap between the 2 groups widened further, with the EBP group scoring 5.7 points higher than the control group (*P* < .001).

**Table 5:** Comparison of self-efficacy between the 2 groups.

Indicator	Control group (n = 42)	Evidence-based practice group (n = 42)	Statistical value	*P* value
GSES score (points, *x̄* ± *s*)				
Pre-intervention (preoperative)	24.5 ± 5.2	23.8 ± 5.6	*t* = 0.596	.553
Postoperative day 3	20.6 ± 4.5	26.2 ± 4.8	*t* = −5.562	<.001
Day of discharge	26.8 ± 4.2	32.5 ± 3.8	*t* = −6.521	<.001

### 3.6. Postoperative complications and safety outcomes

Table 6 compares postoperative complications between the 2 groups. The proportion of patients with at least 1 postoperative complication was 21.4% in the EBP group and 50.0% in the control group (χ^2^ = 7.5, *P* = .006). Abdominal distension (7.1% vs 23.8%) and pulmonary infection (0% vs 9.5%) were less frequent in the EBP group (both *P* < .05). No falls occurred in either group, and other safety events were uncommon.

**Table 6:** Comparison of postoperative complication rates between the 2 groups, n (%).

Complication	Control group (n = 42)	Evidence-based practice group (n = 42)	χ^2^ value	*P* value
Nausea and vomiting	8 (19.0)	5 (11.9)	0.824	.364
Abdominal distension	10 (23.8)	3 (7.1)	4.459	.035
Urinary tract infection (UTI)	4 (9.5)	3 (7.1)	0.157	.692
Urinary retention	5 (11.9)	2 (4.8)	1.424	.233
Pulmonary infection	4 (9.5)	0 (0)	4.200	.040
Unplanned extubation (UE)	1 (2.4)	0 (0)	1.012	.314
Overall complications	21 (50.0)	9 (21.4)	7.500	.006

The overall complication rate represents the proportion of patients with at least 1 listed postoperative complication; patients with multiple complications were counted once in the overall rate.

## 4. Discussion

This retrospective propensity score-matched study compared clinical outcomes among 84 KT recipients receiving either an evidence-based early functional exercise program or conventional care. Receipt of the EBP was associated with earlier 1st ambulation and gastrointestinal recovery, shorter postoperative hospital stay, better ADL and self-efficacy scores, lower pain scores, and a lower observed complication rate. Because the study was observational and retrospective, these findings should be interpreted as associations rather than proof of causal effects.

In this study, the time to 1st postoperative ambulation in the EBP group was approximately 75 hours earlier than that in the control group, and the rate of ambulation within 24 hours increased from 7.1% to 66.7%. This between-group difference may be related to the protocol’s clear initiation timing assessment criteria and phased progressive exercise process. In the conventional care model, healthcare professionals often delay ambulation due to concerns about patient safety, whereas the evidence-based protocol, by specifying concrete initiation criteria (e.g., hemodynamic stability, pain score ≤4), eliminated the ambiguity of subjective judgment, allowing patients to be active as early as possible under safe conditions.^[11,12]^ An association between earlier ambulation and earlier gastrointestinal recovery was also observed in this study, with the EBP group having significantly earlier times to 1st flatus and 1st defecation than the control group. From a physiological mechanism perspective, early activity can stimulate gastrointestinal function recovery by increasing intra-abdominal pressure and promoting intestinal peristalsis, thereby reducing the occurrence of abdominal distension and constipation.^[13]^ This result is consistent with the enhanced recovery after surgery concept, which emphasizes that early activity promotes gastrointestinal function recovery.

In terms of ADL, the BI score at discharge in the EBP group was significantly higher than that in the control group, and nearly half of the patients achieved complete independence in daily living. This difference may be related to the phased structure of the exercise protocol. The evidence-based protocol gradually transitioned from in-bed activities to bedside sitting, standing, and then to indoor walking and stair-climbing training, which aligns with the physiological pattern of muscle function recovery. The daily progressive increase in activity intensity avoided excessive fatigue while continuously stimulating the recovery of muscle strength and joint range of motion.^[14]^ It is worth noting that the independence of the conventional care group showed a significant decline at 3 days postoperatively, which may be related to postoperative pain, fatigue, and fear of activity. In contrast, the EBP group, through early intervention and progressive training, may have mitigated the impact of these factors. From a clinical perspective, rapid recovery of ADL not only reduces the burden of family care but also enhances patients’ confidence in returning to normal life.^[15]^

Regarding postoperative pain, the results of this study did not support the concern that early activity necessarily worsens pain. Although pain scores increased in both groups at 3 days postoperatively compared with preoperative levels, the pain level in the EBP group was significantly milder than that in the control group. Several mechanisms may plausibly contribute to this observation, although they were not directly tested in this study. First, joint movement and muscle contraction in the evidence-based protocol can promote local blood circulation, accelerate the clearance of inflammatory mediators, and thus alleviate pain.^[16]^ Second, regular activity can prevent joint stiffness and muscle spasm, avoiding secondary pain caused by prolonged immobilization. Furthermore, psychological factors also played an important role: patients in the EBP group, having clear rehabilitation goals and a sense of progress, experienced reduced anxiety about pain, and their pain tolerance threshold was correspondingly higher.^[17]^ These findings suggest that, when performed under predefined safety criteria, early functional exercise was not associated with higher observed pain scores in this cohort.

Self-efficacy is an important psychological factor affecting patients’ initiative in rehabilitation. This study found that the self-efficacy score in the control group showed a significant decline at 3 days postoperatively, whereas that in the EBP group increased compared with the preoperative level. This difference may stem from the different experiences of the 2 groups during the rehabilitation process. Patients in the conventional care group lacked a clear exercise plan and progress feedback, easily generating uncertainty such as “Am I doing enough?” or “Is my recovery normal?,” thereby weakening their rehabilitation confidence.^[18]^ In contrast, the evidence-based protocol provided patients with clear staged goals and quantifiable progress indicators (e.g., daily step increments and extended activity duration), enabling patients to intuitively perceive their own progress. This “visible sense of achievement” is a key driver for enhancing self-efficacy.^[19]^ From a clinical practice perspective, improving self-efficacy has important long-term significance – it can translate into intrinsic motivation for continuing functional exercise after discharge, helping patients maintain healthy behaviors and improve long-term prognosis.

In terms of safety, the overall complication rate in the EBP group was less than half of that in the control group, with particularly notable reductions in abdominal distension and pulmonary infection. The incidence of abdominal distension decreased from 23.8% to 7.1%, which may be related to earlier mobilization and its potential effects on gastrointestinal motility. The incidence of pulmonary infection decreased from 9.5% to 0%, which may be related to the deep-breathing and airway-clearance components emphasized in the protocol. These measures maintain airway patency, promote secretion expulsion, and prevent atelectasis and hypostatic pneumonia.^[20]^ It is worth noting that there were no significant differences between the 2 groups in safety indicators such as falls or unplanned extubation, suggesting that, within the limits of this sample, the program was not associated with an observed increase in these safety events when implemented with initiation assessment, vital-sign monitoring, and termination criteria. This finding supports further prospective evaluation of the protocol’s safety and feasibility.

This study has several limitations. First, as a retrospective study, although PSM was used to reduce measured baseline imbalance, unmeasured confounding cannot be completely ruled out. Temporal confounding is possible because surgical care, staffing, perioperative management, or other institutional practices may have changed over time; PSM cannot account for unmeasured secular changes. Second, the data came from a single center with a relatively limited sample size, and the generalizability of the findings needs to be further validated in multicenter, large-sample studies. Third, this study only observed short-term effects during hospitalization and did not follow up on long-term functional status, quality of life, or long-term graft survival after discharge. Additionally, due to the nature of the retrospective study, some detailed data (e.g., specific daily activity duration and detailed records of patient subjective feelings) may have been incompletely documented. Future prospective randomized controlled studies with extended follow-up periods are warranted to further validate the long-term effects of this evidence-based protocol.

In conclusion, in this retrospective propensity score-matched cohort of KT recipients, receipt of an evidence-based early functional exercise program was associated with earlier postoperative recovery, better ADL and self-efficacy, lower pain scores, and a lower observed complication rate. The program may have potential clinical value and appeared feasible under the specified monitoring and termination criteria. Prospective multicenter studies are needed to confirm these associations, assess long-term outcomes, and determine the program’s effectiveness and safety before broad routine implementation.

## Author contributions

**Conceptualization:** Miao Zhang, Peili Shi, Xiaowei Su, Ying Wang, Shuwen Li.

**Data curation:** Miao Zhang, Peili Shi, Xiaowei Su, Ying Wang, Shuwen Li.

**Formal analysis:** Miao Zhang, Peili Shi, Xiaowei Su, Ying Wang, Shuwen Li.

**Funding acquisition:** Shuwen Li.

**Investigation:** Shuwen Li.

**Writing – original draft:** Shuwen Li.

**Writing – review & editing:** Shuwen Li.
